# Prognostic impact of tertiary lymphoid structures in breast cancer prognosis: a systematic review and meta-analysis

**DOI:** 10.1186/s12935-021-02242-x

**Published:** 2021-10-15

**Authors:** Na-Na Zhang, Feng-Jin Qu, Hao Liu, Zhu-Jun Li, Yu-Chi Zhang, Xuan Han, Zi-Yu Zhu, Yi Lv

**Affiliations:** 1 Center for Regenerative and Reconstructive Medicine, Med-X Institute of Western China Science and Technology Innovation Harbour, The First Affiliated Hospital of Xi’an Jiaotong University, Xi’an, 710049 Shaanxi China; 2 National Local Joint Engineering Research Center for Precision Surgery and Regenerative Medicine, The First Affiliated Hospital of Xi’an Jiaotong University, Xi’an, 710061 Shaanxi China; 3 Department of Hepatobiliary Surgery, First Affiliated Hospital, Xi’an Jiaotong University, Xi’an, 710061 Shaanxi China

**Keywords:** Tertiary lymphoid structures, Breast cancer, Prognosis, Immunotherapy, Meta-analysis

## Abstract

**Background:**

Tertiary lymphoid structures (TLSs), organizationally resemble lymph nodes, are frequently present in breast cancer (BCa). It is usually, but not always, associated with a positive prognosis or immunotherapy response in cancer patients. This meta-analysis was performed to assess the prognostic and clinical impact of TLSs in BCa.

**Methods:**

We conducted a systematic search in PubMed, Embase, Cochrane Library, Web of Science, China National Knowledge Infrastructure, and WanFang Database to obtain eligible research data up to May 30, 2021. This meta-analysis is focusing on the studies evaluated the prognostic value of TLSs and the associated clinicopathologic indicators, related gene expression and survival. STATA software 16.0 software was used to assess the prognostic significance and clinical impact of TLSs.

**Results:**

Nine studies involved with 2281 cases were incorporated in this meta-analysis, in which four of them evaluated the prognostic value of TLSs. There are 6 studies assessed the relationship of TLSs and 4 studies investigated the clinicopathologic parameters as well as the key gene expression, respectively. The results showed the presence of TLSs were predicting a better OS (HR = 0.61, 95% CI: 0.51–0.73, *p* < 0.001) and DFS (HR = 0.40, 95% CI: 0.17–0.93, *p* < 0.001) of BCa patients. It also revealed that the presence of TLSs was significantly correlated with tumor differentiation (*p* < 0.001), pTNM stage (*p* < 0.001), lymph node metastasis (*p* < 0.001), and TILs density (*p* < 0.001) of BCa, and the expression of Her2 (*p* < 0.001), ER (*p* < 0.001), PR (*p* < 0.001) and Ki67 (*p* = 0.009) of the tumor cell.

**Conclusion:**

Our results indicated that high levels of TLSs could predict a favorable prognosis for BCa. Moreover, the TLSs were significantly correlated with the clinicopathological indicators and the critical gene expression of BCa, indicating its potential clinical impact on BCa patients.

**Supplementary Information:**

The online version contains supplementary material available at 10.1186/s12935-021-02242-x.

## Introduction

Breast cancer (BCa) is the most commonly diagnosed malignancy in females, imposing pressing scientific, clinical, and societal challenges. The incidence of BCa is rising in most countries and is projected to increase further over the next 20 years despite dramatic efforts for its prevention and treatment. The American Cancer Society has estimated that nearly 30% of new cancer cases and 15% of cancer deaths among women could be caused by BCa [1–3].

Recently, exciting progress in cancer immunotherapy has ushered in a new era in cancer treatment. Immune checkpoint blockade (ICB) therapy has already improved patient survival in various cancers [4, 5]. However, not all cancer patients benefit from immunotherapy. Studies have reported that only approximately 5% of patients with metastatic triple-negative BCa obtain a positive response to PD-1/PD-L1 blockade [6, 7], and the response rate seems to increase to 19–23% upon selection of patients with a PD-L1-positive tumor microenvironment (TME) [8, 9]. Therefore, investigation of the specific factors contributing to the cancer response to immunotherapy and of the corresponding biomarkers predicting the utility and effect of immunotherapy is of great significance for cancer patients [10].

Tertiary lymphoid structures (TLSs) are ectopic lymphoid organs formed in nonlymphoid tissues during chronic inflammation and tumorigenesis. TLSs identified from several solid tumors have been verified to be positively correlated with the response of cancer patients to immunotherapy [11, 12]. In melanoma, the densities of CD20 + B cells and TLSs in ICB responders’ tumor tissues were found to be significantly higher than those in nonresponders [13]. Many studies have indicated that immune cells within TLSs can improve tumor antigen presentation, increase cytokine-mediated signaling, and stimulate CD8 + T cells to attack tumor cells [10, 13]. TLSs are essential sites for initiating and maintaining local and systemic T and B cell responses against tumors. In addition, TLSs are a privileged site for the recruitment of lymphocytes into tumors and the generation of central memory T and B cells, which can circulate and slow cancer progression [14]. Thus, TLSs can be considered a novel biomarker to stratify untreated cancer patients’ survival risk. TLSs are also expected to be a very promising quantitative biomarker for predicting the efficacy of tumor immunotherapy in the future.

Although many studies have reported the positive effect of TLSs on the prognosis of BCa patients [10, 12, 13], the relationships between TLSs and patients prognosis, the expression of associated genes, and other clinicopathological parameters are inconsistent among studies and are even contradictory among samples from patients of different races and detection methods adopted by the researchers [15, 16]. To determine the prospective clinical significance of TLSs, it is essential to clarify the role of TLSs and their relationship with other parameters related to patient prognosis. Thus, this literature-based meta-analysis was conducted to identify a much more specific prognostic value of TLSs and a clear relationship between TLSs and prognosis-related parameters. This comprehensive analysis may provide crucial information on the tumor microenvironment's immune status and its dynamic effect on tumor progression, possibly leading to a more powerful, strengthened biosignature for predicting the clinical outcomes and sensitivity to immunotherapy of BCa patients.

## Materials and methods

### Search strategy

Two authors (NNZ and FJQ) were responsible for independently searching the comprehensive databases and evaluating the availability of studies in PubMed, Embase, the Cochrane Library, Web of Science, China National Knowledge Infrastructure (CNKI) and the WanFang database to obtain eligible studies published before May 30, 2021. The search terms included tertiary lymphoid organ (TLO), tertiary lymphoid structures (TLSs), tertiary lymphoid tissue (TLT), ectopic lymphoid-like structures (ELSs), breast cancer/tumor/malignancy, and prognosis, prognostic or survival outcome. The exact search query is provided as follows to allow reproducibility: (TLO OR TLSs OR TLT OR ELSs) AND (breast cancer OR breast tumor OR breast malignancy) AND (prognosis OR prognostic OR survival outcome). There were no other limitations in the process for searching the databases. Moreover, we reviewed the references in relevant articles to find potential studies. Two researchers independently screened titles and abstracts based on the inclusion and exclusion criteria. Finally, we selected studies that needed reference data through full-text reading. Any differing opinions between the two researchers were resolved through discussion with more researchers.

### Study selection

Original studies eligible for inclusion in this meta-analysis satisfied all of the following criteria: (1) studies focusing on patients with breast cancer (BCa); (2) studies investigating the TLSs in situ in tumor tissue by applying H&E staining and immunohistochemistry; (3) studies assessing the correlation of TLSs with survival or other clinicopathologic indicators and gene expression in BCa; (4) full text, original research article published in English (alternatively, for articles published in Chinese, the English title and abstract were available); and (5) favorable studies selected from similar works published by the same term. The exclusion criteria were as follows: (1) conference abstracts, letters to the editor, reviews, comments, and animal trials; (2) studies with sample sizes < 50, since a small sample size induces publication bias; and (3) work without raw data that could not be traced.

### Data extraction and quality assessment

The incorporated studies' baseline information, including the name of the first author, country of study, publication year, sample size, TLS detection methods, TLS location, grouping methods, follow-up period, and outcome measures, was extracted (Table 1). Additionally, we recorded the original data of the clinicopathologic parameters and related gene expression data for analysis of the relationship with TLSs in a predefined table. The hazard ratios (HRs) and their associated 95% confidence intervals (95% CIs) from multivariate analysis were extracted. The Newcastle–Ottawa Scale (NOS) was used to evaluate the study quality. Two authors (NNZ and FJQ) independently conducted the process and resolved disagreements through discussion with more researchers. The NOS, ranging from 0 to 9, includes three domains: selection of the exposed cohort, comparability of the cohorts, and assessment of the outcome. Studies with a NOS score greater than 6 were considered high-quality studies.

**Table 1:** Main information of the included studies

Author (ref.)	Publishing year	country	No. of patients	TLSs detection methods	TLSs location	Grouping methods	Follow-up (months) Median (range)	Outcome
Figenschau et al. [15]	2015	Norway	290	IHC for T, B cells and HEV	Global	Negative/positive	NA	NA
Kim et al. [17]	2016	Korea	204	IHC for T, B cells and HEV	Tumor adjacent tissues	None, little, moderate, or abundant	NA	NA
Zhou et al. [18]	2016	China	100	IHC for T, B, DC cells, and HEV	Global	Negative/positive	NA	NA
Lee et al. [19]	2016	Korea	769	IHC for T and B cells	Tumor adjacent tissues	None, little, moderate, or abundant	47 years (23–76 years)	OS, DFS
Laurence et al. [20]	2017	Belgium	125	IHC for T and B cells	Global	Negative/positive	NA	NA
Gao et al. [21]	2017	China	150	IHC for T, B, DC cells, and HEV	Global	Negative/positive	NA	NA
Liu et al. [22]	2017	China	248	IHC for T and B cells	Global	Negative/positive	78 (1–134)	DFS
Lee et al. [23]	2019	Korea	335	IHC for T, B cells and HEV	Tumor adjacent tissues	None, little, moderate, or abundant	NA	OS
Chao et al. [24]	2020	China	60	IHC for T, B cells	Tumor adjacent tissues	None, little, moderate, or abundant	48 (22–163)	DFS

*TLSs* tertiary lymphoid structures, *HEV* high endothelial venule, *NA* not available

### Evaluation and collection of clinicopathological parameters and gene expression data for BCa

We traced and collected common clinicopathological data for BCa, including patient age, tumor size, tumor differentiation status, pTNM stage, lymph node metastasis status, lymphovascular invasion status, and TIL density. We collected the original data and divided each cohort into a high and low group for each indicator to ensure the uniformity, rationality, and accuracy of the P values from further Chi-square tests. Similar to the classification standards adopted in most incorporated articles, we classified the BCa patients into a young and an old group according to the cutoff age of 50, a large and a small group (small tumor size ≤ 20 mm, large tumor size > 20 mm), a well-differentiated group (G1 + G2) and a poorly differentiated group (G3 + G4), an early-stage (I + II) group and a late-stage (III + IV) group, and positive and negative lymph node metastasis and lymphovascular invasion groups. Additionally, we grouped patients by TIL density into a low and a high group according to the mean or the median value, whichever was provided, and the patients were also grouped according to the absence or presence of TLSs. Patients were classified by the expression of related genes, including HER2 (human epidermal growth factor receptor 2), ER (estrogen receptor), PR (progesterone receptor), and Ki67, into positive and negative groups. Fisher's method, which combines p values, is appropriate for analysis of this type of data. The original data and *p* values from chi-square tests in the included studies are shown in detail in Additional file 1: Tables S1 and S2, respectively.

### Statistical analysis

Statistical software version 16.0 (Stata Corporation, College Station, TX, USA) was used to perform the analysis, while HR and 95% CI data were log transformed and pooled. The fixed effects model was selected if *I*^2^ < 50%, and the random effects model was considered if significant heterogeneity existed (*I*^2^ > 50%). The heterogeneity among the studies was evaluated by the *I*^2^ statistic proposed by Higgins and the Cochran Q test. The risk of death of BCa patients with a higher density of TLSs was evaluated by HRs and 95% CIs. A HR < 1 indicated a better prognosis. Publication bias was evaluated by visually assessing the asymmetry of an inverted funnel plot and was quantified by Egger’s and Begg’s tests. We combined the *p* values from the chi-square test of each included study by Fisher's method combined with *p* value analysis. *p* < 0.05 indicated that TLSs were statistically related to a certain clinicopathologic parameter or the expression of a certain gene.

## Results

### Study selection and characteristics

**Fig. 1:**
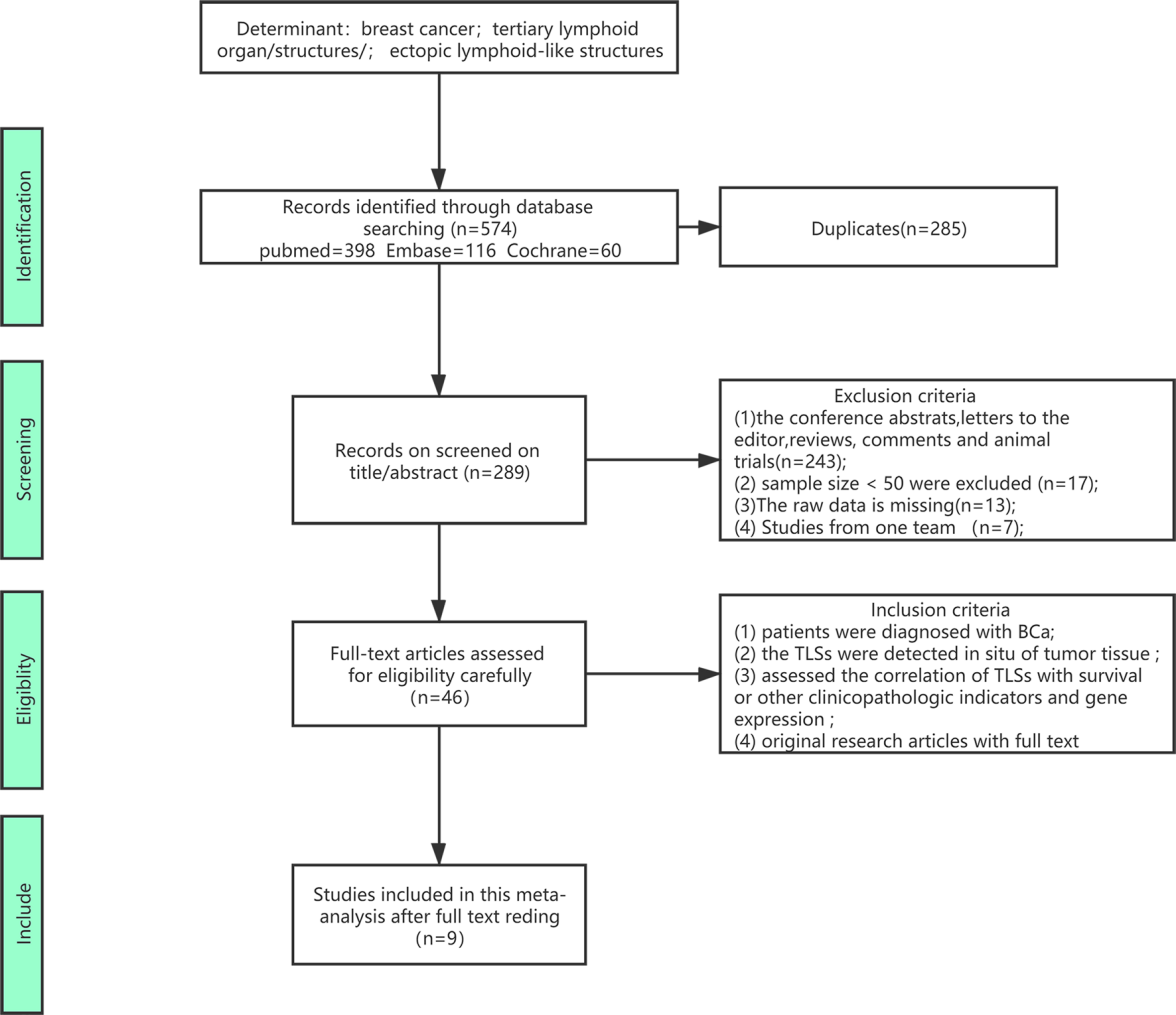
The flow chart of the process of study selection

Using the described search strategy, we identified 574 records in the primary literature search. After screening the titles, abstracts, and methods of each publication, 528 studies were excluded, as they were reviews, animal studies, or irrelevant studies. We carefully assessed the eligibility of each article based on the full text. Finally, 9 studies [15, 17–24] investigating the correlation between TLSs and BCa patient outcomes and including other relevant clinicopathologic parameters or gene expression were included in this meta-analysis comprising 2281 cases. Figure 1 shows the flow diagram for literature retrieval and selection.

**Fig. 2:**
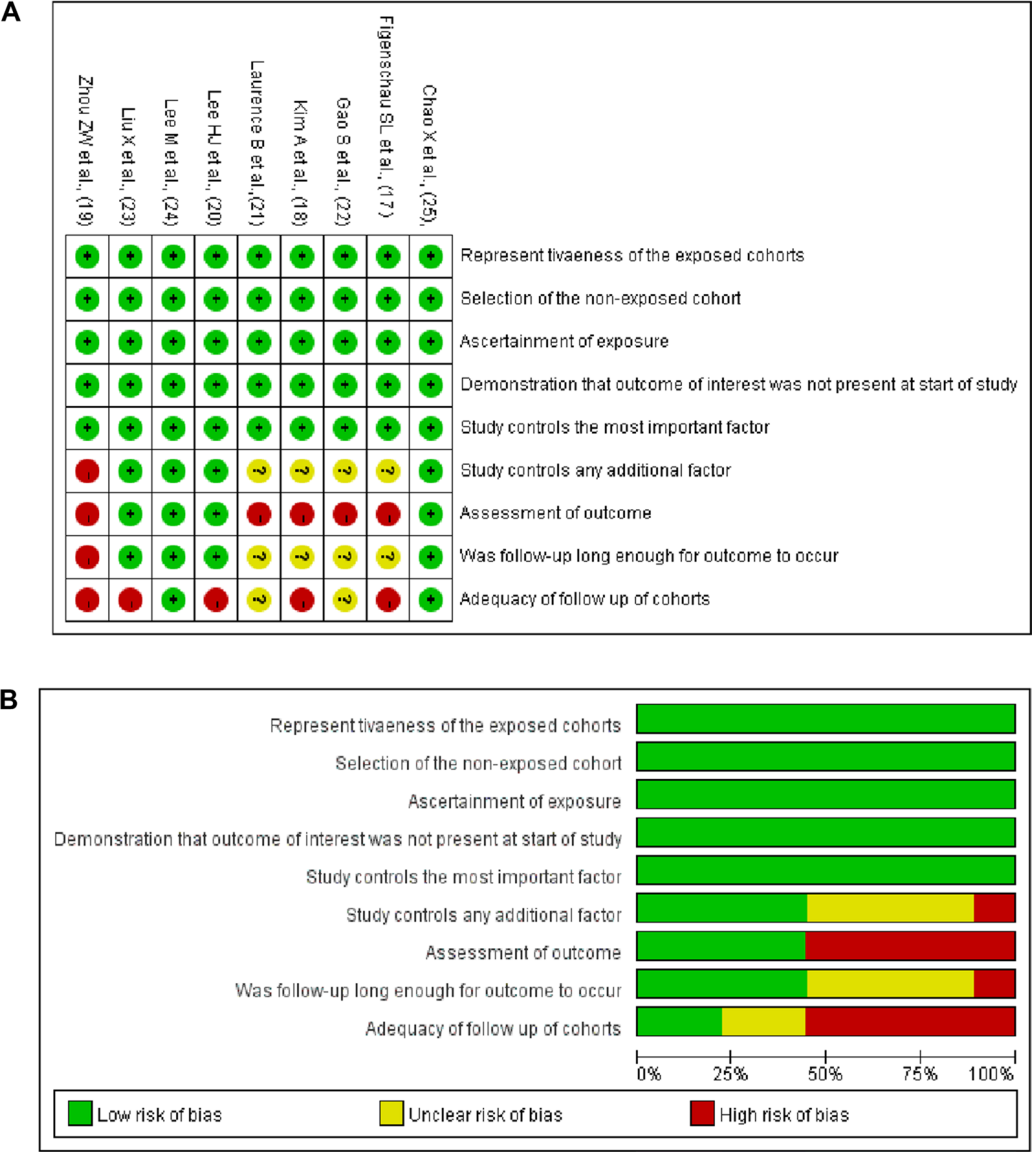
**A** the risk of bias summary plot shows the risk for each of the included studies. The green, yellow, and red point represent low, unclear, and high risk, respectively. **B** the risk of graph bias showed the risk of bias item presented as percentages across all included studies, including increased risk, low risk, and unclear risk

Table 1 summarizes the basic information and main characteristics of the 9 included studies. Among the 9 included articles, 4 studies evaluated the prognostic value of TLSs in BCa: 3 studies evaluated disease-free survival (DFS) [19, 22, 24], and 2 studies evaluated overall survival (OS) [19, 23]. Six studies assessed the relationship of TLSs with other clinicopathologic parameters[15, 17, 18, 20–22]. Four studies included patient age and tumor size [15, 18]; 6 studies included tumor grade [15, 17, 18, 20–22]; 2 studies included pTNM stage [18, 21]; 5 studies included lymph node metastasis status [15, 18, 20–22]; one study included lymphovascular invasion status [22]; and 2 studies included TIL density [20, 22]. Four studies evaluated the association between the expression of related genes and TLSs [15, 17, 20, 22]. Four studies included Her2 expression [15, 17, 20, 22]; 3 studies included ER expression [15, 20, 22]; 3 studies included PR expression [15, 20, 22]; and 2 studies included Ki67 expression [20, 22]. The NOS scores of the included studies ranged from 5 to 9 and are shown in detail in Fig. 2 and Additional file 1: Table S3.

### Prognostic value of TLSs in BCa patients

**Fig. 3:**
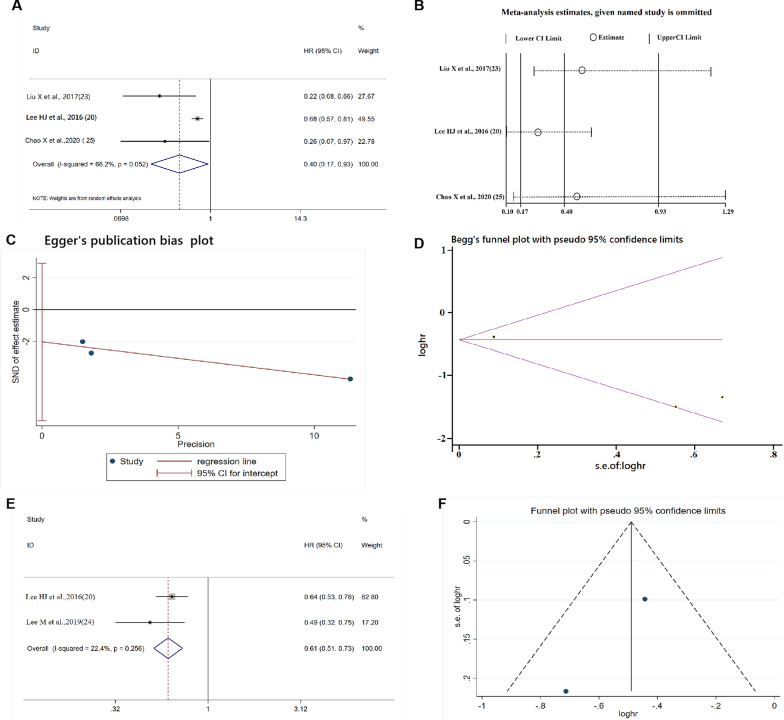
**A**–**D** The forest plot,sensitivity analysis plot, Egger’s test and Begg’s test of the meta-analysis of DFS for BCa patients. **E**, **F** The forest plot and funnel plot of the meta-analysis of OS for the BCa patients, from Cox multivariate analysis

Four studies evaluated the prognostic value of TLSs in BCa [19, 22–24]. Three studies analyzed the prognostic value of TLSs for DFS in BCa [19, 22–24]. The random effects model was adopted: the heterogeneity among the 3 included studies was greater than 50% (Cochrane’s Q, *p* = 0.052, *I*^2^ = 66.2%). The pooled results of the 3 studies comprising 1077 patients revealed that TLSs were significantly correlated with better DFS (HR = 0.40, 95% CI: 0.17−0.93, *p* < 0.001; Fig. 3A). The sensitivity analysis confirmed the pooled results’ stability and credibility, as removal of any cohort failed to change the statistical significance (Fig. 3B). Egger’s and Begg’s tests showed no significant publication bias (*p* = 0.121, *p* = 1.000; Fig. 3C, D), indicating that a higher density of TLSs significantly predicted better DFS for BCa patients.

Only 2 studies [19, 23] evaluated the prognostic value of TLSs for OS in BCa. The fixed effects model was appropriate owing to the lower heterogeneity among the included studies (Cochrane’s Q, *p* = 0.266; *I*^2^ = 22.4%). The results showed that a higher density of TLSs was a favorable prognostic factor for OS in BCa patients (HR = 0.61, 95% CI: 0.51−0.73, *p* < 0.001; Fig. 3E), and the funnel plot in Fig. 3F indicated no publication bias. Information on the analysis of DFS and OS in patients with BCa is shown in Table 2.

**Table 2:** Pooled HR, heterogeneity, and publication bias of the meta-analysis of DFS and OS in BCa

Factor	Include studies	Cases	Model	Pooled HR (95% CI)	*p*	Publication bias		Heterogeneity	
						Egger	Begg	*I*^2^	*p*
DFS	3	1077	R	0.40 (0.17–0.93)	< 0.001	0.121	1.000	66.2%	0.052
OS	2	1104	F	0.61 (0.42–0.90)	< 0.001	–	–	22.4%	0.256

### The association of TLSs with clinicopathological parameters of BCa

Table 3 shows the final results of the correlation analysis between TLSs and various clinicopathological parameters. Four studies [15, 18, 20, 21] analyzed the relationship between TLSs and patient age, and the results indicated no significant correlation between TLSs and patient age (χ^2^ = 5.332, *p* = 0.722). Similarly, no significant correlation was found in the 4 studies [15, 18, 20, 21] that analyzed the relationship between TLSs and tumor size (χ^2^ = 4.149, *p* = 0.843). Tumor differentiation status (χ^2^ = 70.765, *p* < 0.001), pTNM stage (χ^2^ = 19.690, *p* < 0.001), lymph node metastasis status (^χ2^ = 31.939, *p* < 0.001), and TIL density (χ^2^ = 27.631, *p* < 0.001) were found to be significantly correlated with TLSs. One paper [22] reported the relationship between TLSs and lymphovascular invasion without finding statistical significance (χ^2^ = 3.647, *p* < 0.056, Additional file 1: Table S1).

**Table 3:** The fisher's method combine p-value analysis of the correlation between TLS and various clinicopathological parameters and gene expression

Parameters	No. of cohorts	Number of cases	χ^2^ value	p	Relationship with TLSs
Patients’ age	4	665	5.332	0.722	No statistical significance
Tumor size	4	665	4.149	0.843	No statistical significance
Tumor differentiation	6	1117	70.765	< 0.001	Negative
pTNM stage,	2	250	19.690	< 0.001	Negative
Lymph node metastasis	5	913	31.939	< 0.001	Negative
TILs density	2	373	27.631	< 0.001	Positive
HER2	4	867	42.448	< 0.001	Positive
ER	3	663	33.098	< 0.001	Negative
PR	3	663	35.455	< 0.001	Negative
Ki67	2	373	13.468	0.009	Positive

### The association of TLSs and related gene expression of BCa

We also investigated the expression of key genes (HER-2, ER, and PR) affecting the diagnosis, therapy selection, and prognosis of BCa to evaluate the relationship of these genes with TLSs. A total of 4 papers [15, 17, 20, 22], 3 papers [15, 20, 22] and 3 papers [15, 20, 22] reported the association of HER-2, ER, and PR, respectively, with TLSs. Positive correlations were found between these genes (HER-2, χ^2^ = 42.448, *p* < 0.001; ER, χ^2^ = 33.098, *p* < 0.001; PR, χ^2^ = 35.455, *p* < 0.001) and TLSs. Two articles [20, 22] documented the relationship between Ki67 (reflecting the tumor proliferation index) and TLSs, and the analysis results showed that TLSs were also significantly correlated with Ki67 expression (χ^2^ = 13.468, *p* = 0.009, Table 3).

## Discussion

Recent studies have highlighted the role of TLSs in the immunotherapeutic response of malignant tumors [12, 13]. In general, the presence of TLSs is an indicator of favorable prognosis in many solid tumors. However, some works have reported a negative prognostic impact of TLSs. Previous studies reported that the presence of TLSs was significantly associated with a more advanced stage in colorectal cancer [25] and a higher tumor grade, as well as a higher frequency of lymph node metastasis, in BCa [15]. These inconsistent results may be due to the nonuniform evaluation methods adopted in these studies. Therefore, we assessed the prognostic role and clinicopathological impact of TLSs in this meta-analysis incorporating 9 different cohorts containing a total of 2281 patients with BCa. Although only 4 studies evaluated the prognostic value of TLSs, the presence of TLSs was found to be an independent favorable prognostic factor for OS and DFS in BCa, indicating that the TLS status in tumor tissue is essential for predicting the clinical outcomes of BCa. Previous literature has recorded that “TLSs could provide a favorable milieu for the education of intratumoral CD8 + T cells and B cells against cancer”[11]. Generally, TLSs represent sites of lymphoid neogenesis characterized by mature dendritic cells in a T cell zone adjacent to a B cell follicle including a germinal center. Recently, investigators developed a semantic segmentation model for whole-slide histopathological images, named HookNet, which can segment TLSs and germinal centers in lung cancer [26]. Inflammatory BCa (IBC) is an aggressive form of this disease, and it shows higher expression of TLS signatures with higher sensitivity to immune checkpoint inhibitors [27]. Therefore, the formation of TLSs indicates a vigorous rather than an exhausted immune response. Attempts to induce the formation or modulate the function of TLSs may offer new therapeutic opportunities for cancer patients.

Recent advances in tumor immunology have shown that the tumor microenvironment is in a state of dynamic evolution [28]. The complex interactions among immune and tumor cells are crucial for tumor progression [29]. A recent study reported that lymphocyte clusters (LCs), like TLSs, are closer to tumor cell islands in patients with a good outcome, with a decrease in the LC size with decreasing distance to tumor cell islands [30]. Thus, it is crucial to identify the clinicopathological impact of TLSs in BCa. This meta-analysis first analyzed the relationships between TLSs and other clinicopathological indicators. Our results showed that TLSs were not significantly correlated with patient age or tumor size but markedly influenced the clinicopathological indicators that reflected tumor progression (e.g., tumor differentiation status, pTNM stage, lymph node metastasis status, and lymphovascular invasion status), suggesting that TLSs may be closely related to tumor progression and evolution. In addition, we found that the density of TILs displayed a positive correlation with the absence of TLSs. A study in colorectal cancer reported that TLSs were linearly associated with lymphocyte infiltration. TLSs provide a pathway for recruitment of TILs and cooperate with TILs in a coordinated antitumor immune system response, predicting low-risk, early-stage colorectal cancer [31].

It is well known that the development of BCa is strongly associated with multiple gene aberrations. Molecular analysis can enhance the understanding of the biological behavior of BCa. The key molecules, HER-2, ER, and PR, have been found to play a critical role in the diagnosis, therapy selection, monitoring, and prognosis of BCa. Thus, the correlations between these critical molecules and TLSs were analyzed in the present meta-analysis, and significant correlations were identified. It was found that the monoclonal antibody trastuzumab was associated with a complete or partial response in patients with HER-2-positive tumors, characterized by higher infiltration of leukocytes and an augmented capacity to mediate antibody-dependent cellular cytotoxicity, compared with patients with nonresponding tumors [32]. TLSs have been reported to be associated with significantly better DFS in HER-2 + IBC but not in HER-2− IBC [22]. Since tumoral TLSs can promote more effective antitumor immunity [33, 34], and an active immune response is important for treatment of HER-2 + IBC, it was documented that many HER-2 + BCa patients are treated with chemotherapy and/or HER-2 targeted therapy and that some of the favorable effects are attributed to active antitumor immunity [35–37]. Passive immunotherapy, particularly with trastuzumab and pertuzumab, is an effective therapeutic strategy in HER2 + BCa [38]. Therefore, the presence of TLSs might be an indicator of the treatment response in Her2 + BCa patients. And one study explored the association of TLSs and drug responses in BCa patients, but not directly explored their relationship. The study found a higher frequency of TLSs in IBC than non-IBC, and the IBC were more sensitive to ICIs than non-IBC [27]. It indicates that a higher frequency of TLSs in BCa tissue might be vulnerable to checkpoint inhibitors (ICIs). Another study found TLSs in the BCa were infiltrated with PD-L1 + , PD-L2 + , LAG3 + , and TIM3 + (immune checkpoint molecule) cells, which has an important implication for the successful response to the immunotherapy [39]. These observations suggest that TLSs are important site of immune activation and regulation, particularly in tumors with extensive baseline immune infiltration. While whether TLSs can predict the effect of immunotherapy is worthy of further exploration and validation by the large sample clinical trials.

ER and PR are used as indicators to evaluate prognosis and guide clinical endocrine therapy in BCa. An in silico analysis of gene expression profiles in 2976 nonmetastatic BCa samples showed that ER- and PR-positive BCa tissues were infiltrated with their characteristic immune components and directly affected the prognosis of BCa patients [40]. Cell proliferation is a basis for tumorigenesis, and Ki67, a nuclear antigen associated with proliferating cells, is an important marker for effectively evaluating the proliferative activity of tumor cells [41]. TLSs were also significantly correlated with Ki67 expression, although only two documents reported this connection. In addition, the advent of new genetic tests has emphasized the role of Ki67 as a prognostic and predictive marker in BCa. Specialists have reassessed evidence that could change guidelines to include Ki67 in the standard pathological assessment of BCa [42]. The summary of these analyses revealed significant correlations between TLSs and various indicators, reflecting tumor biological behavior and affecting tumor diagnosis, therapy selection, monitoring, and prognosis. Notably, accurate detection of the number of TLSs is based on large pathological tissues, since extensive sampling can allow detection of more morphological structures. Studies have proven that intratumoral TLSs are an indicator of favorable prognosis in patients with pancreatic cancer [43], while peritumoral TLSs correlate with protective immunity and improved prognosis in patients with hepatocellular carcinoma [44], indicating the significance of TLSs localization in cancer patients. However, information on the specific histomorphological location is more dependent on extensive sampling; thus, larger pathological tissues imply a higher accuracy of morphological information and are of great importance for predicting the prognosis of patients.

## Conclusions

In summary, TLSs have attracted great interest among researchers in the field of tumor immunology. Our analysis results indicated that a high level of TLSs was significantly correlated with favorable outcomes for BCa patients. In addition, there were significant correlations among the clinicopathological indicators, expression of critical genes and presence of TLSs. Thus, TLSs are expected to be a biomarker for predicting prognosis and guiding immunotherapy in BCa and have great significance for the development of individualized treatment.

## Supplementary Information

**Additional file 1: Table S1**. The original data and chi-square test for the TLSs and clinicopathologic parameters. **Table S2** The original data and chi-square test for the TLSs and related gene expression. **Table S3.** NOS scores of included studies.

## Data Availability

Not applicable.

## Abbreviations

TLSs: Tertiary lymphoid structures
BCa: Breast cancer
IBC: Inflammatory breast cancer
OS: Overall survival
DFS: Disease-free survival
ICB: Immune checkpoint blockade
ICIs: Checkpoint inhibitors
LCs: Lymphocyte clusters
TILs: Tumor infiltrating lymphocytes
TMEs: Tumor microenvironments
HER2: Human epidermal growth factor receptor 2
ER: Estrogen receptor
PR: Progesterone receptor
