# Supplementary material for: Prognostic impact of tertiary lymphoid structures in breast cancer prognosis: a systematic review and meta-analysis
Source: Cancer Cell Int. 2021 Oct 15;21:536. doi: 10.1186/s12935-021-02242-x (PMC8520238; doi:10.1186/s12935-021-02242-x)
Supplement: Supplementary file 1 — Additional file 1: Table S1. The original data and chi-square test for the TLSs and clinicopathologic parameters. Table S2 The original data and chi-square test for the TLSs and related gene expression. Table S3. NOS scores of included studies. [file 12935_2021_2242_MOESM1_ESM.doc]

**Table S1 the original data and chi-square test for the TLSs and clinicopathologic parameters**

| Author and publishing year | TLSs | Age | χ2(P) | Tumor size | χ2( P) | Grade | χ2( P) | pTNM stage | χ2( P) |
| --- | --- | --- | --- | --- | --- | --- | --- | --- | --- |
|  |  | Young old |  | Small Large |  | G1+G2 G3+G4 |  | I+II III+IV |  |
| Figenschau SL et al., 2015 ^17^ | Absent | 59 137 | 4.424  (0.35) | 99 57 | 0.509（0.475） | 139 16 | 19.203  (＜0.001) |  |  |
|  | Present | 21 89 |  | 56 39 |  | 64 31 |  |  |  |
| Kim A et al., 2016 ^18^ | Absent |  |  |  |  | 76 48 | 61.534  (＜0.001) |  |  |
|  | Present |  |  |  |  | 5 75 |  |  |  |
| Zhou ZW et al., 2016^19^ | Absent | 23 21 | 0.059（0.808） | 10 34 | 0.627（0.429） | 29 15 | 4.104  （0.043） | 29 15 | 3.740  (0.053) |
|  | Present | 16 13 |  | 9 20 |  | 26 3 |  | 25 4 |  |
| Laurence B et al., 2017^21^ | Absent | 11 38 | 0.379（0.538） | 28 21 | 0.002（0.966） | 35 14 | 6.611  (0.010) |  |  |
|  | Present | 20 53 |  | 42 31 |  | 35 38 |  |  |  |
| Gao S et al., 2017^22^ | Absent | 40 48 | 0.553（0.457） | 36 52 | 0.222（0.638） | 41 47 | 33.021  (＜0.001) | 35 53 | 45.359  (＜0.001) |
|  | Present | 32 30 |  | 23 39 |  | 57 5 |  | 59 3 |  |
| Liu X et al.,2017^23^ | Absent |  |  |  |  | 90 65 | 16.828  (＜0.001) |  |  |
|  | Present |  |  |  |  | 29 64 |  |  |  |

Table S1 (continued)

| Author and publishing year | TLSs | lymphnode metastasis | χ2( P) | Lymphovascular invasion | χ2( P) | TILs density | χ2( P) |
| --- | --- | --- | --- | --- | --- | --- | --- |
|  |  | Negative Positive |  | Negative Positive |  | Low High |  |
| Figenschau SL et al., 2015 ^17^ | Absent | 130 45 | 3.660(0.056) |  |  |  |  |
|  | Present | 70 40 |  |  |  |  |  |
| Zhou ZW et al., 2016^19^ | Absent | 16 28 | 5.953(0.015) |  |  |  |  |
|  | Present | 19 10 |  |  |  |  |  |
| Laurence B et al., 2017^21^ | Absent | 32 17 | 0.003(0.959) |  |  | 37 6 | 18.056(＜0.001) |
|  | Present | 48 25 |  |  |  | 29 35 |  |
| Gao S et al., 2017^22^ | Absent | 25 63 | 34.972(＜0.001) |  |  |  |  |
|  | Present | 48 14 |  |  |  |  |  |
| Liu X et al., 2017^23^ | Absent | 76 75 | 2.134（0.144） | 124 31 | 3.647（0.056） | 131 20 | 24.921(＜0.001) |
|  | Present | 37 54 |  | 63 28 |  | 48 35 |  |

Table S2 the original data and chi-square test for the TLSs and related gene expression

| Author and publishing year | TLSs | Her2 expression | χ2( P) | ER expression | χ2( P) | PR expression | χ2( P) | Ki67 expression | χ2( P) |
| --- | --- | --- | --- | --- | --- | --- | --- | --- | --- |
|  |  | Negative Positive |  | Negative Positive |  | Negative Positive |  | Negative Positive |  |
| Figenschau SL et al., 2015 ^17^ | Absent | 141 14 | 14.734  (<0.001) | 10 144 | 29.184  (<0.001) | 20 82 | 16.672  (<0.001) |  |  |
|  | Present | 69 26 |  | 31 64 |  | 36 38 |  |  |  |
| Kim A et al.,2016 ^18^ | Absent | 74 10 | 48.207  (＜0.001) |  |  |  |  |  |  |
|  | Present | 49 74 |  |  |  |  |  |  |  |
| Laurence B et al., 2017 ^21^ | Absent | 42 7 | 0.265  （0.606） | 5 44 | 3.396  （0.065） | 8 41 | 5.427  （0.020） | 35 14 | 7.371  （0.007） |
|  | Present | 60 13 |  | 17 56 |  | 26 47 |  | 34 39 |  |
| Liu X et al.,2017 ^23^ | Absent | 132 22 | 15.456  (＜0.001) | 43 111 | 12.158  (＜0.001) | 45 108 | 15.048  (＜0.001) | 134 19 | 1.880  （0.170） |
|  | Present | 59 33 |  | 46 46 |  | 50 42 |  | 73 17 |  |

Table S3 NOS scores of included studies

| **Author (ref)** | **Year** | **Selection** | | | | **Comparability** | | **Outcome** | | | **Nos score** |
| --- | --- | --- | --- | --- | --- | --- | --- | --- | --- | --- | --- |
|  |  | **Item 1^a^** | **Item 2^b^** | **Item 3^c^** | **Item 4^d^** | **Item 5^e^** | **Item 6^f^** | **Item 7^g^** | **Item 8^h^** | **Item 9^i^** |  |
| Figenschau SL et al., ^17^ | 2015 | 1 | 1 | 1 | 1 | 1 | ? | 0 | ? | 0 | 5 |
| Kim A et al., ^18^ | 2016 | 1 | 1 | 1 | 1 | 1 | ? | 0 | ? | 0 | 5 |
| Zhou ZW et al., ^19^ | 2016 | 1 | 1 | 1 | 1 | 1 | ? | 0 | ? | 0 | 5 |
| Lee HJ et al.,^20^ | 2016 | 1 | 1 | 1 | 1 | 1 | 1 | 1 | 1 | 1 | 9 |
| Laurence B et al.,^21^ | 2017 | 1 | 1 | 1 | 1 | 1 | ? | 0 | ? | ? | 5 |
| Gao S et al., ^22^ | 2017 | 1 | 1 | 1 | 1 | 1 | ? | 0 | ? | ? | 5 |
| Liu X et al., ^23^ | 2017 | 1 | 1 | 1 | 1 | 1 | 1 | 1 | 1 | 1 | 9 |
| Lee M et al., ^24^ | 2019 | 1 | 1 | 1 | 1 | 1 | 1 | 1 | 1 | 1 | 9 |
| Chao X et al., ^25^ | 2020 | 1 | 1 | 1 | 1 | 1 | 1 | 1 | 1 | 1 | 9 |

^a^ Item 1: Representativeness of the exposed cohorts

^b^ Item 2: Selection of the non-exposed cohort

^c^ Item 3: Ascertainment of exposure

^d^ Item 4: Demonstration that outcome of interest was not present at start of study

^e^ Item 5: Study controls the most important factor

^f^ Item 6: Study controls any additional factor

^g^ Item 7: Assessment of outcome

^h^ Item 8: Was follow-up long enough for outcome to occur

^i^ Item 9: Adequacy of follow up of cohorts
